# Computed tomography image analysis of cobalt oxide nanoparticles-induced cytopathic effect on Hep-G2 cell lines

**DOI:** 10.1039/d5ra05722a

**Published:** 2025-12-10

**Authors:** Mohamed S. Nasr Eldin, Moustafa A. Soula, Ahmed A. Emara, Hala M. Ahmed, Widad M. Al-Bishri, Gharieb S. El-Sayyad

**Affiliations:** a Radiology and Medical Imaging Department, Faculty of Applied Health Sciences Technology, October 6 University 6th of October City Giza Egypt; b Medical Imaging and Radiography Department, Faculty of Allied Medical Sciences, Aqaba University of Technology Aqaba Jordan; c Biomedical Equipment Department, Faculty of Applied Health Sciences Technology, October 6 University 6th of October City Egypt; d Department of Biological Sciences, College of Science, University of Jeddah Jeddah 80327 Saudi Arabia; e Medical Laboratory Technology Department, Faculty of Applied Health Sciences Technology, Badr University in Cairo (BUC) Cairo Egypt Gharieb.El-Saied@buc.edu.eg

## Abstract

The main aim of the present study is to examine the effect of cobalt oxide nanoparticles (Co_3_O_4_ NPs) on the proliferation of hepatocellular carcinoma (Hep-G2) cells. The cells were exposed to red light with a wavelength of *λ* = 655 nm for 30 minutes at a power of 50 mW cm^−2^ and in the dark. The sol–gel method was used to create Co_3_O_4_ NPs, which were then calcined at 800 °C. HRTEM, XRD, SEM, FTIR, DLS, and zeta potential tests were used to examine Co_3_O_4_ NPs and to determine their shape, size, crystal structure, surface appearance and charge, and size range. The results revealed that light-induced photodynamic treatment is highly detrimental. MATLAB-R2010a and Fiji (ImageJ) open-source program powered by the National Institutes of Health (US) software were used to analyze the collected data to determine its quantitative or qualitative qualities through the use of gray-level histogram analysis. After the tested cells were looked at in grayscale images made with computed tomography (CT) and Co_3_O_4_ NPs, we found that there was a broad peak in the middle. On the other hand, the histogram shows a leftward skew and shift with a peak that has a wide base and a sharp top or bottom in cell lines. This study provides strong evidence that Co_3_O_4_ NPs can stop Hep-G2 cell lines from multiplying. The data from CT images of Co_3_O_4_ NPs showed a high degree of statistical significance. This work summarizes the use of metallic NPs, particularly Co_3_O_4_ NPs, in CT imaging to detect and treat cancer.

## Abstract 2

This work summarizes the use of metallic Co_3_O_4_ NPs, in CT imaging to detect and treat the proliferation of hepatocellular carcinoma (Hep-G2) cells.

## Graphical abstract

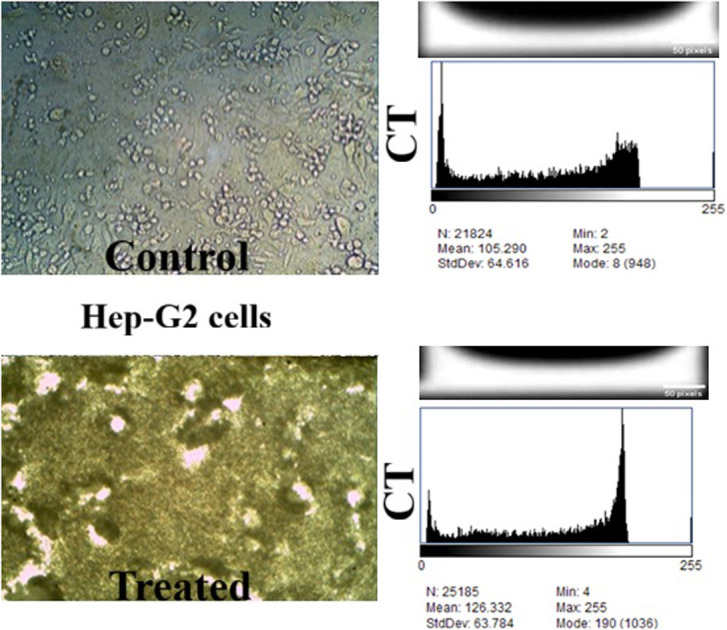

## 1. Introduction

One of the greatest common diseases that impact people's health globally is cancer.^1^ Chemotherapy is the most widely used treatment for cancer because of its potential effectiveness; yet, it has serious side effects, including hair loss, constant fatigue, mouth soreness, and dry, itchy, or irritated skin.^2^ Some anticancer medications do not work as well as they used to.^3^ Additionally, misuse of antibiotics caused multidrug resistant microorganisms to arise globally.^4^ Therefore, there is a great need to find novel chemicals with anticancer properties. A majority of researchers in many sectors, including medical, pharmaceutical, agricultural, environmental, and industrial fields, have focused their attention on nanotechnology and nanomaterials.^5^

Numerous investigations on nanostructured substances have paved the way to the development of novel functional material categories with distinctive characteristics and uses.^6^ Tiny crystalline metal oxides have garnered increasing attention recently because of their large surface areas, distinct adsorptive qualities, and quick diffusivities.^7^ Transition metal oxides, such as Co_3_O_4_ NPs, are P-type semiconductors. They may exist in several oxidation states, such as Co^2+^, Co^3+^, and Co^4+^.^8^ Numerous physical and chemical methods, such as coprecipitation, microwave-assisted, chemical-sprayed pyrolysis, thermal decomposition, solution the combustion process, sonochemistry, the microemulsion, and hydrothermal processes, have been used to produce Co_3_O_4_ NPs.^9,10^

Cobalt oxide is one type of nanoparticle that has shown great promise as a material for a variety of modern technological uses, such as information storage and the creation of sophisticated machinery and systems.^11,12^ Nano-sized Co_3_O_4_ are particularly appealing due to their special structural, catalytic, magnetic, and electrical capabilities.^13^ Interestingly, because of their large surface area, Co_3_O_4_ NPs have increased chemical and physical reactivity, making them appropriate for catalytic activities.^11,14^ Certain physical and chemical features must be taken into account for the use of Co_3_O_4_ NPs in a variety of cutting-edge fields and modern sectors, such as analytical and separation innovations, data and information storage systems, catalysis, biological medicine, and nanoparticles.^15^ These characteristics include being distinct, having the same dimensions and form, displaying a homogeneous crystal structure, and preserving a constant composition.^16^

Cobalt oxide is a transition metal that is necessary for the proper functioning of plants, animals, and insects.^17^ It is possible to find a wide variety of inorganic complexes displaying various analyses, including oxidation states. It is important to remember that not all forms of oxidation are harmful to health.^18^ The material in concern is a crystalline molecule that dissolves in water and breaks down when electrolyzed.^19^

Among other physiological activities, Co_3_O_4_ NPs play a significant role in the synthesis of white blood cells, red blood cells, and immunological responses.^20^ Additionally, it is essential for the synthesis of DNA and vitamin B-12, which supports the growth, development, and maintenance of newborns' nervous systems.^21,22^ Furthermore, studies have indicated that it may have neuroprotective properties and improve renal and cardiovascular health.^23^

Because Co_3_O_4_ NPs cluster in tumors, nanotechnology, and pathogenic cells, and since they are energized by light that is visible of an appropriate wavelength that forms free radicals as well as ROS, resulting in cell death, the succeeded in determining the photodynamic effectiveness of Co_3_O_4_ NPs had been determined in the following ref. 24 and 25.

Nanoparticles of cobalt oxide are useful in medicine because they have special properties that make them antifungal, antibacterial, antioxidant, anticancer, larvicidal, anticholinergic, antileishmanial, wound healing, and anti-diabetic agents.^25–30^ Co_3_O_4_ NPs, which have useful magnetic properties, are used a lot in biomedical tasks like magnetic resonance imaging, magnetic hyperthermia, and magnetic targeting.^31^ Co_3_O_4_ NPs has a very large surface area, which gives it unique electrical, optical, catalytic, and magnetic properties.^32^ This makes it a good candidate for biological applications. Cobalt nanoparticles in different oxidation states (*i.e.*, Co^2+^, Co^3+^, and Co^4+^) are advantageous in many applications. Co_3_O_4_ NPs work as catalysts to speed up the process of turning hydrogen peroxide (H_2_O_2_) into harmful hydroxyl radicals (˙OH), which are bad for tumor cells.^33^ Still, it is possible to increase the production of reactive oxygen species (ROS) and cure cancer by mixing these Co_3_O_4_ NPs with drugs or even more nanoparticles.^34^

In clinical practice, computed tomography (CT) has become an essential imaging modality.^35^ It was the first technique to take noninvasive pictures of the human body without being influenced by the assumption of certain anatomical.^36^ This results from the information being projected into a two-dimensional scanning plane, as is commonly observed in planar X-ray fluoroscopy. As a result, compared to traditional radiography, CT produces pictures with substantially better contrast.^37^

This study proves beyond a doubt that Co_3_O_4_ NPs have either proliferative or antiproliferative effects on Hep-G2 cell lines. The graphs from all the areas of interest found in the CT images of Co_3_O_4_ nanoparticles for both the Hep-G2 and control groups, made with Fiji (ImageJ), show that our results are very important.

## 2. Materials and methods

### 2.1. Cell lines and cell culture

The human hepatocellular carcinoma cells Hep-G2 ATCC 8065 cell lines were procured from VACSERA, located in Giza, Egypt. Hep-G2 cells were subcultured in a 75 cm^2^ flask using DMEM media supplemented with penicillin (Biochrom), streptomycin (Biochrom), 2 mM l-glutamine (Biochrom), and 10% heat-inactivated fetal bovine serum (FBS) (HyClone, UK). The cells were incubated at 37 °C in a humidified atmosphere containing 5.2% CO_2_ until a confluent monolayer was observed. Hep-G2 cells at aconcentrateion of 1.5 × 10^5^ cells per mL.

The cells were then maintained in an exponential growth state.^38^ The infant flask bottles that had been inoculated were subjected to daily microscopic observation over seven days to identify any variations, such as cellular alterations in morphology and the formation of a cytopathic effect (CPE). The flasks that exhibited a CPE were subjected to a freeze-thaw process, whereby they were frozen and then thawed three times.^39^ Well plates were filled with 1–20 µg mL^−1^ solutions of distributed Co_3_O_4_ NPs, which were then exposed to red light at a wavelength of 655 nm (50 mW cm^−2^) for 30 minutes. The Co_3_O_4_ NPs uptake was then allowed to incubate for 24 hours.^40^ ISO 10993-5:2009 for Biological Evaluation of Cytotoxicity Testing For assessing cytotoxicity of Co_3_O_4_ NPs on Hep-G2 cells had been followed.

### 2.2. Synthesis and characterization of Co_3_O_4_ NPs

We bought ethanol (40 mL mol^−1^, 99.9% purity), oxalic acid (99.9% purity), and cobalt nitrate salt (99.9% purity) from Sigma-Aldrich Chemicals in Egypt. The molar ratio of cobalt nitrate to ethanol to water was determined to be 1.5 : 1 : 5, after cobalt nitrate salt was dissolved in distilled water and ethanol, 1.6 g of oxalic acid was dissolved in water and stirred constantly for 20 minutes. At room temperature (24.0 ± 2 °C), and the pH was checked and found to be slightly acidic (6.1). The resulting gel was aged for 24 hours, dried for 2 hours at 120 °C, then sintered at 800 °C to prepare the resulting nano-powder to further analysis.^41^

The mean size distribution of the produced Co_3_O_4_ NPs was ascertained by means of dynamic light scattering measurements, utilizing the DLS-PSS-NICOMP 380-ZLS particle-sized apparatus at the St. Barbara, California, USA facility. A temporary test sample of 100 µL of Co_3_O_4_ NPs was placed in a small cuvette. Five operations were conducted after two minutes of stabilization at an ambient temperature of 25.0 ± 2 °C.

Crystallinity and crystal size estimates were assessed using XRD (XRD-6000, Shimadzu Research Instruments, Japan). The generated Co_3_O_4_ NPs were evaluated for size, shape, and overall appearance utilizing a high-resolution transmission electron microscope (HR-TEM, JEM2100, Jeol, Japan). Using a scanning electron microscope (SEM, ZEISS, EVO-MA10, Germany), the surface morphology of the synthetic Co_3_O_4_ NPs was investigated. The surface charges of the created Co_3_O_4_ NPs were measured at the pH they were made at, using a zeta potential analyzer from Malvern Device, UK, and the experiment was done three times. Finally, the FT-IR analysis was an important strategy that provided information on the type of link between Co_3_O_4_ NPs and oxalic acid, as well as the chemical functional groups that corresponded to the synthesized Co_3_O_4_ NPs.

### 2.3. *In vitro* toxicity assay

The use of Co_3_O_4_ NPs in toxicity investigations was assessed using the Mosmann colorimetric test.^42^ The experiment used 96-well plates with a flat bottom to cultivate cell cultures. In each well, 50 µL of cell lines were introduced and incubated in an environment containing 5.2% CO_2_ at 37.5 °C for one night. Subsequently, a volume of 50 µL of culture media containing Co_3_O_4_ NPs at 60 °C, previously dissolved in Dulbecco's Modified Eagle Medium (DMEM), was introduced into each well to achieve a final 1 mg mL^−1^ concentration.

The cell lines were present in each well at a final concentration of 1.5 × 10^5^ cells per mL. The medium had a 10% fetal bovine serum (FBS) concentration. The plates were subjected to incubation in a 5.2% carbon dioxide environment at a temperature of 37.5 °C for 72 hours. Subsequently, a 10-µL aliquot of a sterile solution containing tetrazolium salts (MTT) at 5 mg mL^−1^ concentration in sterile phosphate buffer saline (PBS) was introduced into each well.

The plates were incubated in an environment containing 5.2% CO_2_ at 37.5 °C for 3 hours. The culture medium was meticulously removed, and 100 µL per well of dimethyl sulfoxide (DMSO) was introduced after that. The physical characteristics' optical density (OD) was measured using the MRX Revelation Dynex Technologies microplate reader at a wavelength of 650 nm, which served as the reference point in the enzyme-linked immunosorbent assay.

Each concentration of Co_3_O_4_ NPs was examined in triplicate, with three tests being done. The quantity of Co_3_O_4_ NPs that resulted in a 50% drop in viable cells, as measured by MTT absorbance, was determined by using the formula 1 − OD (sample)/OD (control) × 100, where OD represents optical density. This calculation was performed in comparison to untreated control samples. The plates were subjected to daily microscopic observation for three days to identify and assess any cellular alterations and the emergence of cytopathic effects (CPE).

### 2.4. Photo-induced toxicity

After a 24-hour incubation period, the MTT test and tetrazolium reduction assay were used to assess the cells' viability. The following formula was utilized to determine the percentage viability:^43^
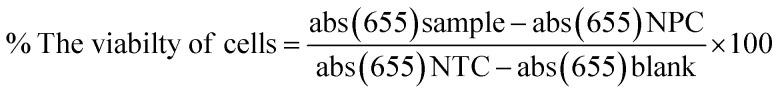


The wells were filled with new culture media containing no nano conjugate for the non-treated positive group that was the control.

### 2.5. Radiological modalities

Radiological modalities are considered a constantly expanding domain within contemporary medicine, including medical imaging, clinical applications, research environments, and advancements in the area.^44^

Medical imaging plays a crucial role in the identification, initial diagnosis, and early detection of many diseases.^45^ Additionally, it aids in selecting optimal treatment strategies, facilitates surgical procedures, and enables monitoring of treatment outcomes, among other applications.^46^

The CT equipment used in this study was the Philips MX16Evo CT scanner. The scanning technique was conducted in the Radiology Department of the October 6 University Hospital, located in October City. Axial cuts were made on the phantom during the scanning process.

### 2.6. Quantitative analysis of CT images

Fiji (ImageJ) open-source program from the National Institutes of Health in the US is a quantitative tool was used to do a quantitative analysis of the CT images. A Region of Interest (ROI) was manually selected for each CT image (for example, with a rectangle selection tool) to cover the area of the cell culture that showed the highest uptake of cobalt oxide nanoparticles (Co_3_O_4_ NPs) or cytopathic effects (or the control area).

Histogram was used to detect the spread out of pixel intensities in the region of interest ROI. The histogram provided us with the Mean Pixel Intensity, Standard Deviation (StdDev), Minimum and Maximum Intensity, and Mode. This histogram data was based on the 8-bit grayscale range of 0–255. This method guarantees scientific rigor and complete repeatability of the quantitative imaging outcomes.

### 2.7. Validation measurements

The validation measurements were conducted using the open source software program Fiji (ImageJ), developed by the National Institutes of Health (NIH), USA. quantitatively analyze the collected CT image data and extract relevant metrics for comparison between experimental groups.

The sensitivity and specificity of a diagnostic test refer to the proportions of cell lines that are confirmed to have an abnormality or illness and correctly test positive or negative for it, respectively. The sensitivity may be expressed as follows:
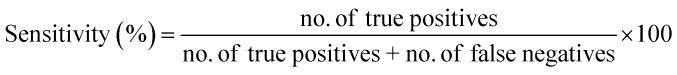


Specificity is related to the test's ability to identify negative data or results. This can be given by:
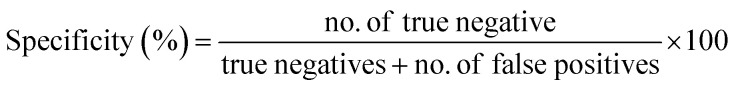

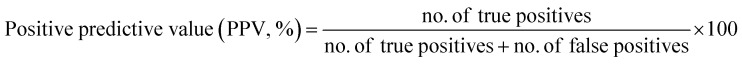

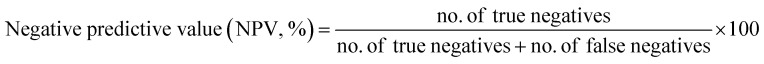


A significant sensitivity of 100% indicates that the test detects all genuine positives, such as all cases are identified as being changes. Therefore, negative findings in a high-sensitivity test indicate out the condition, in contrast to various measures in a high-specificity test.^47^ The mean ± standard deviation (SD) was used to measure and determine continuous parameters or variables. Tukey's test and the analysis of variance (ANOVA) *F*-test were used to determine if the values observed in the various groups differed statistically significantly (*P* < 0.05).

### 2.8. Statistical analysis

To record continuous variables or traits, the mean ± SD was employed. Tukey's test was used after an ANOVA-*F* test to determine the significance of differences between all groups (*P* < 0.05). In order to compute statistical values and measures produced from the gray-scale brightness levels of the image pixels, the histogram analysis employs a variety of techniques and processes. The mean ± standard error (SE) was the data or values. The statistical program SPSS-12 for Windows (Chicago, IL, USA) was used to analyze the data, and a significance level of *P* < 0.05 denoted statistical significance.

## 3. Results

### 3.1. Characterization of the synthesized Co_3_O_4_ NPs

A Cu-Kα (*λ* = 1.54 Å) was used to measure all XRD patterns using a P'analytical X′Pert-PRO diffractometer. All compositions' *hkl* planes are precisely indexed to those found in the standard JCPDS card no. 073-1701,^48^ which deals with Co_3_O_4_ NPs that have a spinel cubic form and the *cF*56 space group. After using the Scherrer formula, 81 nm was discovered to be the crystallite size of the nanoparticles that were calcined at 800 °C which found 18 nm.

**Fig. 1:**
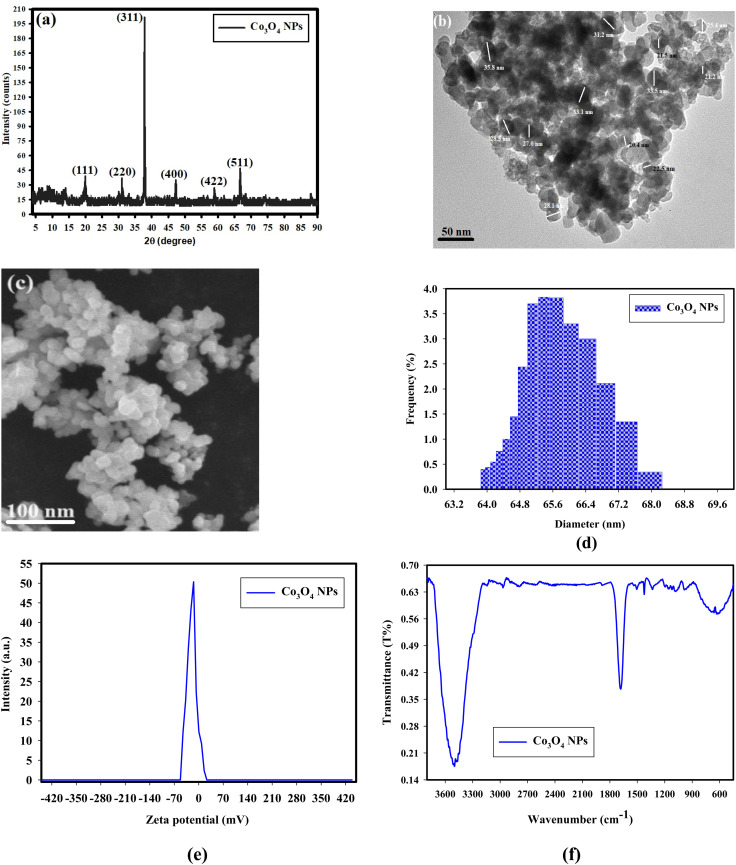
Characterization of the synthesized Co_3_O_4_ NPs, where (a) X-ray diffraction patterns, (b) HRTEM imaging, (c) SEM imaging, (d) DLS analysis, (e) zeta potential, and (f) FTIR analysis.

The Co_3_O_4_ NPs diffraction peaks at 2*θ* = 19.58°, 31.87°, 37.85°, 47.09°, 58.48°, and 67.80°, as shown in Fig. 1a, represent the angles (111), (220), (311), (400), (422), and (511), respectively, of Bragg's reflections. These peaks are confirmed by the standard card JCPDS number 073-1701.^48^

The size and form of the produced Co_3_O_4_ NPs were characterized by HR-TEM analysis (Fig. 1b). The average size of each particle could be found, and the produced Co_3_O_4_ NPs could be observed. As can be observed in the HR-TEM image, the generated Co_3_O_4_ NPs come in a range of morphologies, namely round and spherical ones. The diameter range for Co_3_O_4_ NPs is displayed in Fig. 1b, ranging from 21.2 nm to 35.8 nm, with an average particle size (APS) as 27.35 ± 2.0 nm.

The anisotropy form had been documented in that study,^49^ however even while diverse morphologies may be seen due to the manufactured procedure from extract, the formed forms may vary as the extracted NPs' shapes were always generally circular or ellipsoidal. One cap and reducing agent is used in our experiment, which results in a consistent form.

The produced Co_3_O_4_ NPs, which also turned out to be dazzling particles, are shown in Fig. 1c. The produced Co_3_O_4_ NPs' SEM confirmations are displayed as clustered NPs with a sphere-like shape. A review of the literature regarding morphological shape and elemental analysis revealed that the generated Co_3_O_4_ NPs (in the current work) were evenly distributed, broad in size, and had the same spherical shape. DLS analysis was performed to determine the particle size distribution (PSD) of Co_3_O_4_ NPs in order to evaluate the dispersion of particle sizes. The findings are given in Fig. 1d as 65.39 nm.

It is typical for DLS size examinations to develop greater values when compared with HR-TEM measurements because DLS evaluation evaluates the hydrodynamic dimension of NPs attached by molecules in water (a solvent), leading to larger dimensions of the wrapped NPs, and HR-TEM evaluation determines the exact particle size of the material without solvent layer.^50^ Because of the scientific reliability of DLS, the NPs that were created were extensively distributed in a limited range of sizes, which greatly enhanced their properties and uses,^51^ but the exact size must be determined by using the HRTEM.

As illustrated in Fig. 1e, the zeta potential of the produced Co_3_O_4_ NPs was assessed during their synthesis at a pH of 6.1. According to the present findings, the zeta potential of the produced Co_3_O_4_ NPs stay negative at the pH of the material throughout testing. Additionally, as illustrated in Fig. 1e, the zeta potential found to be −14.65 mV during the preparation at a slightly acidic pH of 6.1. The different sizes, distributions, and zeta potential values of the generated Co_3_O_4_ NPs are listed in Table 1.

**Table 1:** PSD, APS, and zeta potential values of the synthesized Ag–Se NPs

Nanomaterial	PSD; DLS (nm)	APS; HRTEM (nm)	Zeta potential (mV)
Co_3_O_4_ NPs	65.39	27.35	−14.65

As shown in Fig. 1f, the FTIR analysis was carried out to determine the interaction between the produced Co_3_O_4_ NPs, and oxalic acid functional groups. The fingerprint band (610.32 and 580.86 cm^−1^) is complex because of overlapping vibrations (C–O, and C–N). The FTIR spectrum of Co_3_O_4_ NPs-incorporated oxalic acid showed absorption peaks at 3499 cm^−1^ for –OH stretch, 1685 cm^−1^ for –C=O stretch of ketone groups, and 1329 cm^−1^ for –C–O group.^52^ Furthermore, the prominent peaks seen at 638 cm^−1^, might also be the result of cobalt metallic NPs interacting and combining with hydroxyl groups of oxalic acid to generate Co–O.^53^

### 3.2. Cellular viability

**Fig. 2:**
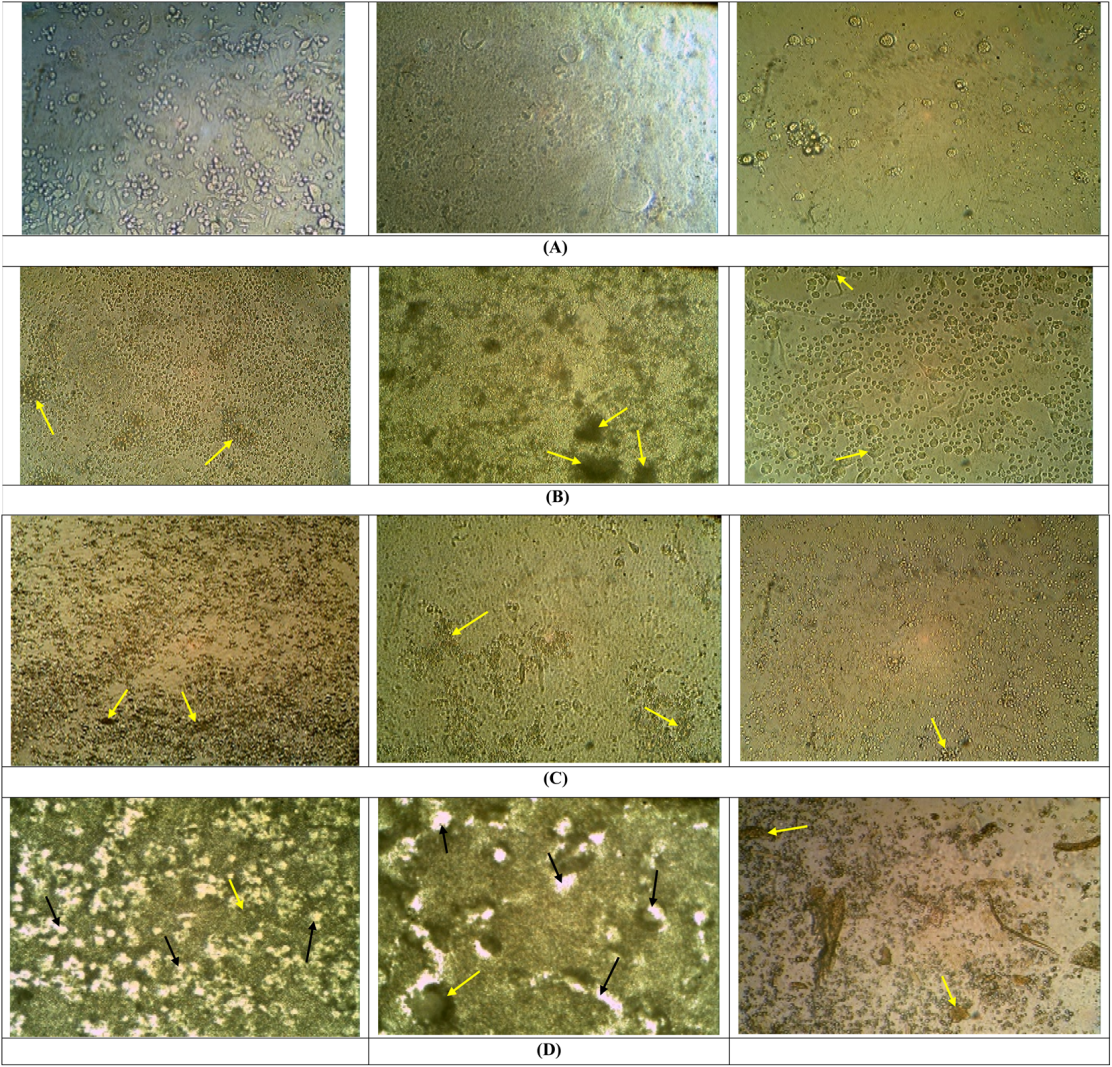
Shows harvested Hep-G2 cells without and with the treatment of Co_3_O_4_ NPs, where (A) control, (B) displays the morphological changes in cell activity after incubation with Co_3_O_4_ NPs for 24 hours, (C) displays the morphological changes in cell activity after incubation with Co_3_O_4_ NPs for 48 hours, and (D) displays the morphological changes in cell activity after incubation with Co_3_O_4_ NPs for 72 hours. (yellow arrows for cell shrinkage, black arrows for creation of vacuoles).

Fig. 2 displays the morphological changes in cell activity after incubation with Co_3_O_4_ NPs for 24, 48, and 72 hours in comparison with control group. These changes were concentration-dependent, differing from the normal cell lines or controls. The cells exhibited several indications of morphological alteration or cellular demise, characterized by distinct deformations and alterations in membrane integrity. These changes included the loss of intercellular contact, vacuolation, cellular rounding, and detachment from the culture plates (Fig. 2).

The morphological changes seen in Fig. 2 indicate the harmful effects of Co_3_O_4_ NPs on Hep-G2 cell lines. However, the treatment did not elicit a favorable response, as shown in Fig. 2. The morphological characteristics of Hep-G2 cells after treatment with Co_3_O_4_ NPs are examined. As the results show, the picture displays distinct and observable spherical cells detached from the culture plate. The creation of vacuoles (shown as black arrows in B, C, and D), cell shrinkage (shown as yellow arrows in B, C, and D), and a decrease in cell population indicate reduced cell proliferation and an increase in cell death. The manifestation of cellular demise is notably more pronounced among groups B, C, and D. The experimental conditions are as follows: (A) control group (untreated); (B) experimental group after 24 hours; (C) experimental group after 48 hours (first iteration); (D) experimental group after 72 hours. Observable alterations in cellular morphology are evident in the control group or normal cells compared to the groups subjected to treatment.

Table 2 presents the experimental evaluation of the proliferative and antiproliferative effects of Co_3_O_4_ NPs on the Hep-G2 cell line. Due to its notable efficacy as both a proliferative and antiproliferative drug, Co_3_O_4_ NPs was subsequently investigated for its effects on Hep-G2 cell lines.

**Table 2:** Presents the results of the inhibition of Hep-G2 cell lines, both those treated and those not treated with Co_3_O_4_ NPs

Hours	Groups	Inhibition (%)	Mean value ± SD	Coefficient of variation	Maximum value	Minimum value
Control	Control	34	109.79 ± 66.45	0.605	194.0	4.00
24	Exposed	41	112.53 ± 66.80	0.593	193.0	4.00
48	Exposed	66	106.03 ± 63.57	0.599	193.0	3.00
72	Exposed	79	109.45 ± 65.61	0.599	193.3	3.66

Table 3 presents the inhibitory effects of Co_3_O_4_ NPs on the fast growth of cancer cells and its ability to produce maximal cell death in Hep-G2 cells. The IC_50_ values for Co_3_O_4_ NPs in Hep-G2 cells were determined to be 8.45, 30.54, 44.89, and 70.81 mg L^−1^, respectively.

**Table 3:** Presents the inhibitory results, precisely the IC_50_ values, of Hep-G2 cell lines that were either treated or not treated with Co_3_O_4_ NPs^a^

Hours	Groups	Inhibition (%)	Regression equation	IC_50_	*F*-ratio
Control	Control	34	*Y* = 22.23 ln(*x*) − 30.2, *R* = 0.97***	49.2	488.09***
24	Exposed	41	*Y* = 14.25 ln(*x*) + 25.2, *R* = 0.87***	8.23	1366.3***
48	Exposed	66	*Y* = 23.23 ln(*x*) − 17.22, *R* = 0.999***	21.92	45.09***
72	Exposed	79	*Y* = 36.66 ln(*x*) − 53.93, *R* = 0.988***	29.33	16.09***

a R***P < 0.001; ***P < 0.001; *n* = 5.

The toxicity of Co_3_O_4_ NPs was higher in cancer cell lines than in normal cell lines. Co_3_O_4_ NPs was discovered to possess significant efficacy as an antiproliferative agent, leading to its further investigation against Hep-G2 cell lines as shown in Tables 4, and 5.

**Table 4:** The data are compared between the groups according to different variables using MATLAB-R2010a

Hours	Groups	The specificity (%)	The sensitivity (%)	Mean value ± SD	Coefficient of variation	Maximum value	Minimum value
Control	Control	79.5	87.5	66.45 ± 64.78	−0.49	189.0	4.00
24	Exposed	83.3	90.0	65.76 ± 64.54	−0.50	192.0	4.00
48	Exposed	62.5	69.2	63.61 ± 66.30	−0.54	191.0	4.00
72	Exposed	73.0	87.5	65.28 ± 65.21	−0.51	190.6	4.00

**Table 5:** The average quantitative histogram parameters (mean, mode, and standard deviation and mode) extracted from CT images of Hep-G2 cells at various exposure intervals following exposure using the Fiji (ImageJ) program

Hours	Groups	The specificity (%)	The sensitivity (%)	Mean	StdDev	Minimum value	Maximum value	Coefficient of variation	Mode
0	Control	77.5	97.46	110.75	64.03	1.33	255	0.578	65
24	Exposed	90.0	70.37	117.91	66.46	1	235	0.563	189
48	Exposed	90.0	70.37	125.96	65.07	0	255	0.517	191
72	Exposed	69.23	73.07	122.58	64.98	3.33	255	0.53	129

The table demonstrates the average values of CT histogram parameters for Hep-G2 cells exposed to cobalt oxide nanoparticles and red light (655 nm, 50 mW cm^−2^) under photodynamic conditions across several time intervals (0 h, 24 h, 48 h, and 72 h) and describes the mean intensity that increases progressively from the control group (110.75) to a peak at 48 h (125.96), followed by a slight decline at 72 h (122.58) while mode values show a similar pattern, rising sharply from 65 in the control group to 191 at 48 h, then decreasing to 129 at 72 h and provide insight into pixel intensity clustering the higher mode values at 24 h and 48 h indicate a concentration of pixels at higher intensity levels, consistent with nanoparticle aggregation and cellular stress. The decreasing at 72 h suggests structural breakdown and loss of uniformity, the Standard Deviation remains relatively stable (≈64–66), indicating consistent variability in pixel intensity distribution. And the Coefficient of Variation (CV) decreases from 0.578 at 0 h to 0.517 at 48 h, then slightly increases to 0.530 at 72 h. This pattern reflects reduced heterogeneity during peak nanoparticle accumulation, followed by increased variability as cellular structures begin to degrade. The detected balance between specificity and sensitivity aligns with the changes in intensity as exposure time increases, signal strength tends to increase, while classification accuracy shows variability. This pattern may indicate biological adaptation or potential constraints in the detection algorithm under different intensity distributions.

### 3.3. Effect of photodynamic exposure on pixel intensity and cytopathic progression

Hep-G2 cells were exposed to red light at a wavelength of 655 nm and an intensity of 50 mW cm^−2^ for 30 minutes under both illuminated and dark conditions. The histogram analysis over time demonstrated a gradual and quantifiable elevation in mean intensity and a corresponding shift in mode values, supporting the hypothesis that Co_3_O_4_ nanoparticles elicit time-dependent cytopathic effects potentiated by photodynamic mechanisms. The observed changes in CT image brightness and histogram distributions align with light-facilitated cellular damage and enhanced nanoparticle-mediated stress responses.

### 3.4. Graphical comparison between groups

**Fig. 3:**
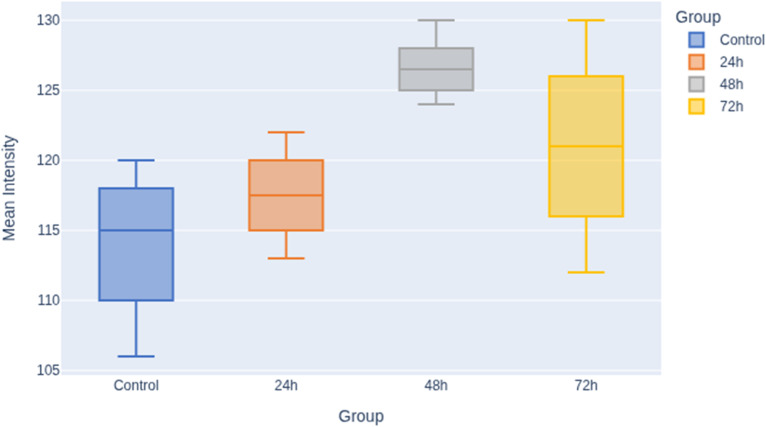
Boxplot shows a clear upward in mean pixel intensity values with increasing exposure time. The control group shows the lowest median and wider variability, 24 h group shows a moderate increase. The 48 h group demonstrates a more consistent elevation in intensity, and the 72 h group demonstrates the highest median with a broader distribution.

The following graph illustrates the comparison of mean intensity values between the experimental groups (control, 24 h, 48 h, and 72 h) as shown in Fig. 3.

## 4. Discussion

People have long used heavy metals and their complexes because of their advantageous physical and chemical characteristics.^54^ Recently, researchers have explained the mechanism and method of metal ions operating inside cancer cells.^55^ It is well known that Co_3_O_4_ NPs can create conditions similar to hypoxia both in *in vivo*^56^ and *in vitro*.^57^ Faraji N. *et al.*,^58^ found that Co_3_O_4_ NPs and menthol can effectively fight colorectal cancer cells, likely by causing cell death and changing the activity of genes related to cell death processes. However, Mahmoudi A.,^59^ found that Co_3_O_4_ NPs covered with glucose and connected to ellagic acid can stop liver cancer cells from growing by raising oxidative stress, blocking the cell cycle, and causing cell death. Finally, as noted by Tajmehri H.,^60^ Co_3_O_4_ NPs covered in glucose and connected to lapatinib can block growth signals and start processes that lead to cell death, helping to stop the growth of lung cancer cells. Table 6 shows how the results of this study relate to earlier research on the impact of other metals-based nanocomposites and their oxide NPs on different cell lines, and their relation to CT imaging.

**Table 6:** Shows how the results of this study relate to earlier research on the impact of other metals-based nanocomposites and their oxide NPs on different cell lines

No.	The synthesized NPs	Synthetic method	Reaction mechanism	Ref.
1	2-Mercaptosuccinic acid (MSA)-coated gold (Au) NPs	Fen's technique to create Au NPs, which served as both a capping and reducing agent	Au NPs are frequently employed in imaging and treatment because of their surface plasmonic characteristics. Their optical activity was confirmed by the plasmonic absorption, which varied between 510 and 540 nm. When compared to Au NPs the CT imaging with MSA-Au NPs was superior. This advantage was associated with Au NPs's increasing surface area as its diameter shrank. These characteristics of Au NPs showed that they were appropriate for multimodal imaging and delivery applications	61
2	PEGylated Cu_2−*x*_Se NPs	An ambient aqueous method	The addition of Cu and Se elements allowed for improved CT imaging both *in vitro* and *in vivo*. The reticuloendothelial organs were where these NPs gathered most, indicating that renal clearance was the source of the fleeing body. After 16 days of therapy, the tumor completely vanished and never returned. It wasn't until 40 days following treatment that the animals' principal organs showed signs of inflammation or injury	62
3	Gold nanoflowers stabilized by dendrimers with extremely small iron oxide NPs (Fe_3_O_4_/Au DSNFs)	A seed-mediated method	Within 60 minutes of the injection, the tumor's CT value had grown by 1.7 times. The CT signal intensity exceeded that of the surrounding tissues, further demonstrating its enhanced localized imaging capabilities. Within an hour of injection, the reticuloendothelial track finally removed the NPs. Ninety-six hours after injection, all of the main organs were completely clear of NPs	63
4	Silver sulphide nanoparticles (Ag_2_S NPs)	Simple reduction method	When given to female naked mice, Ag_2_S NPs' clearance capability and dispersion profile demonstrated a greater bloodstream endurance than that of iodine (control). The bladder showed a significant increase in CT contrast *in vivo*, but the spleen and liver displayed a modest rise. The circulation half-life of Ag_2_S NPs was 86.6 minutes. We then use urine to eliminate them	64
5	Bismuth (Bi) NPs modified with polyethylene glycol (PEG)	A one pot synthesis strategy	There was no discernible hemolysis from the Bi-PEG NPs. After 30 minutes, the principal organs were evident in the mouse model, and Bi-PEG's distribution efficiency was outstanding. Additionally, after an hour, more organs started to show up on CT scans, and at this point, the complete liver was clearly apparent. The liver fully absorbed the material after three hours. The experiment proved that Bi-PEG had a lengthy circulation time, which was essential for CT imaging based on contrast	65
6	Tungsten oxide NPs (WO_3_) coated with polycaprolactone (PCL)	Simple reduction method	They had a high CT number, showed no symptoms of toxicity *in vivo*, and were removed a few hours after ingestion. Although PCL-WO_3_ NPs were removed quickly, they had a longer blood circulation time of up to two hours (rather than ten minutes for iodinated contrast agents). They were a good fit for CT angiography as they did not build up in the body and provided less of a long-term risk	66

Microscopic studies of Hep-G2 cells treated with Co_3_O_4_ NPs have shown that their shape has changed in many ways.^67^ For example, apoptotic bodies have formed, chromatin condensation has changed, and cells have shrunk or broken.^68^ The changes in the shape and structure of Hep-G2 cells show that they are moving toward apoptotic cell death.^69^ Our research found that some things, like the amount of Co_3_O_4_ NPs present, were linked to changes in the activity of cells in Hep-G2 in a way that depended on the dose.^70^ The Hep-G2 cell line was utilized to evaluate the impact of the synthesized Co_3_O_4_ NPs calcined at 800 °C on the toxicity and cellular survival.^71^ HepG2 cells were grown in a dish and put in the dark.^72^ They were also shown red light (with a wavelength of 655 nm) for 30 minutes at a power of 50 mW cm^−2^.

The digital memory is equipped with a 512 × 512 fixed-size image matrix memory, corresponding to the dimensions of the columns and rows of digital picture components, also known as pixels.^73^ Each pixel inside a 512 × 512 matrix corresponds to a distinct shade within the gray-scale spectrum.^74^ The machines kept all pictures or cases in the image memory storage section of the digital scan converter.^75^ To find all the instances in a picture's histogram, you have to keep track of the different locations and frequencies where each pixel's power intensity falls within a certain range in the picture.^76^ In the case of an eight-bit picture, the histogram encompasses eight bits, resulting in a range of gray-scale levels from 0 to 255, totaling 256 values.^77^

Similarly, for a 12-bit image, the histogram will include twelve bits, resulting in a range of 212 gray-scale levels, which equates to 4096 values.^78^ This pattern continues for higher bit depths. The digital scan converter saved all the pictures or data it got from each method and phase as 8-bit files, which means they could have any of 255 grayscale levels.^79^

The digitized photos underwent offline processing by transferring them to a personal computer (PC) for histogram analysis.^80^ This analysis was carried out using two software packages, Fiji (ImageJ) open-source program and MATLAB-R2010a, which served as image analysis tools.^81^ The program MATLAB-R2010a facilitates matrix manipulation, data and function visualization, differentiation, algorithm implementation, and the development of user interfaces.^82^ These programs are developed in several programming languages, such as FORTRAN, C, and C^++^.^83^ Differentiation and comparison of various cell lines were achieved using MATLAB-R2010a Image and Fiji (ImageJ) packages and their respective processing toolboxes.^84^

Our results states that Fiji (ImageJ) was used for initial histogram analysis due to its widespread availability and user-friendly interface. We considered it suitable for extracting pixel intensity distributions within defined regions of interest (ROI). And it is a specialized tool for quantitative biomedical image analysis.^85,86^

**Fig. 4:**
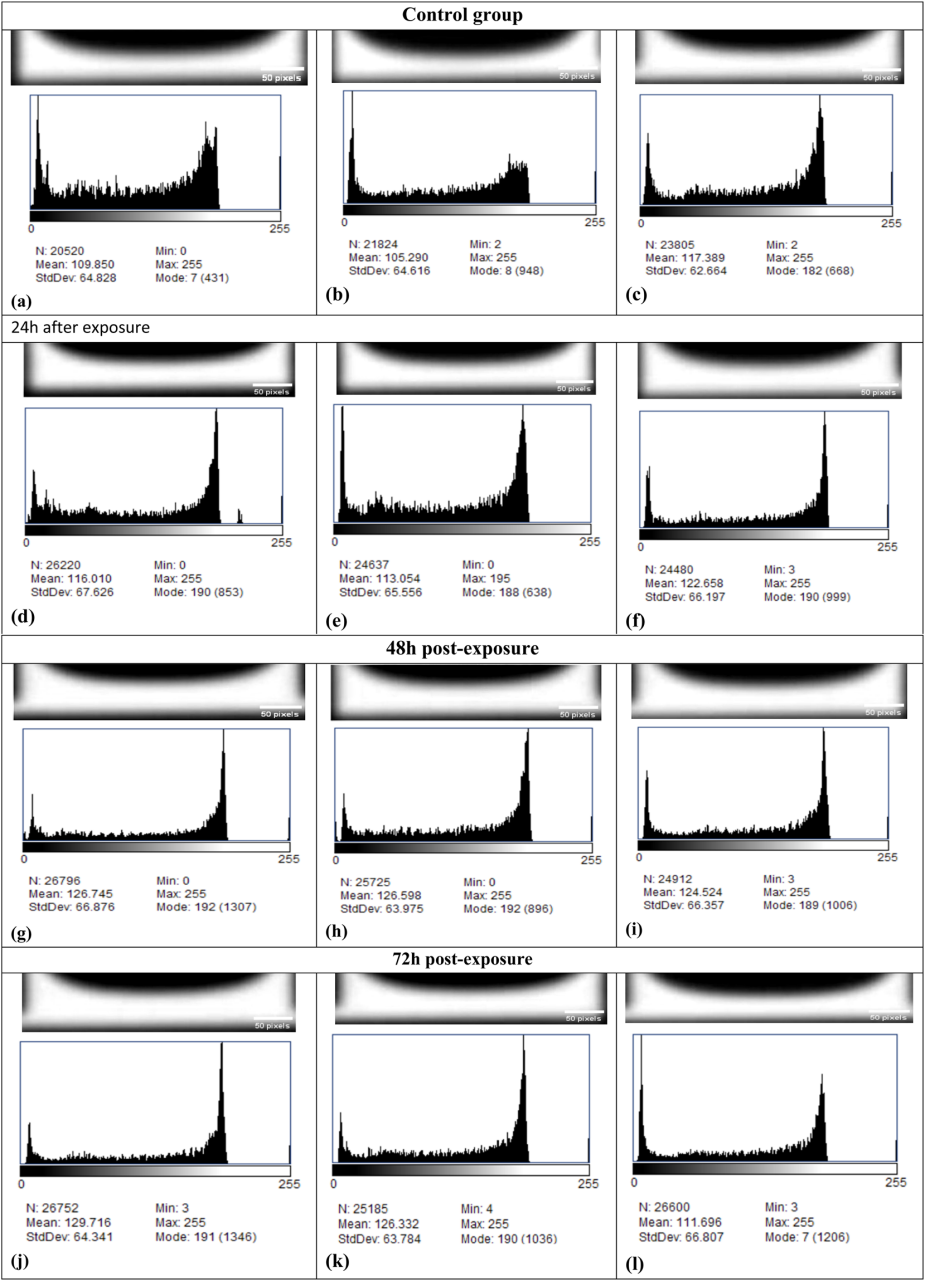
(a–c) Demonstrates the control group, which contain the untreated Hep-G2 cells demonstrates a mean pixel intensity values ranging from 105.29 to 117.39, and mode values ranges between 7 and 182, indicates a minimal intracellular density and absence of nanoparticle accumulation and they served as a standard by which others may be measured. (d–f) 24 h after exposure demonstrates the first cellular responses to Co_3_O_4_ nanoparticles and red light (655 nm, 50 mW cm^−2^) under photodynamic conditions twenty-four hours after exposure at which the mean intensity values increased to 113.05–122.67, and mode values shifted to 188–190, reflecting initial intracellular uptake of Co_3_O_4_ nanoparticles and the onset of cytopathic alterations. (g–i) 48 h after exposure, demonstrated a further elevation in mean intensity (124.52–126.75) and a consistent mode of 190–192, highlighting progressive nanoparticle accumulation and increased cellular stress, leading to a more pronounced cytopathic impact. (j–l) At 72 hours after exposure, the increased mean values (126.33–129.72) and mode values near 190–191, confirming pronounced cytopathic effects. Interestingly, image (l) showed a reduction in both mean (111.70) and mode (7) values, which may indicate late-stage cell death or degradation, resulting in loss of internal density and collapse of structure.

Our study used CT scans of Co_3_O_4_ NPs from both the control group and the Hep-G2 group to make the histograms of the regions of interest (ROIs). We created these scans using Fiji (ImageJ). It is important to note, though, that the histograms were made after the ROIs were chosen, and their descriptive properties were calculated at the same time, as shown in Fig. 4.

The present work describes that CT imaging was performed on *in vitro* tissue samples obtained from biological specimens or experimental tissues. It clarifies that the samples were biological tissues immersed in a medium suitable to simulate physiological conditions. The imaging was not performed *in vivo* on live animals but on prepared tissue specimens to evaluate tissue characteristics post-treatment. The specific parameters of the CT scanner used are detailed, and the purpose was to assess structural changes in the samples. This description aligns with standard *in vitro* imaging procedures in biomedical research.^87,88^

In histogram processing, ROIs were manually delineated in the affected tissues using Fiji (ImageJ). Histograms were generated with specific settings, normalized to baseline intensity, and thresholded at specific value to exclude noise.

Histogram variables frequently served as descriptors for the form and profile of histograms. The selection of these individuals was based on the results of similar studies that used histogram parameters to differentiate different types of tumors.^89,90^ To make both quantitative and qualitative comparisons, the statistical features of the grayscale values inside the ROIs are used to measure intensity or parameters.^91,92^ Upon analyzing the attributes of specificity and sensitivity, it is clear that an enhancement in sensitivity results in a reduction in specificity and *vice versa*.^93^

According to the present findings, the reproducibility and validation of the results by independent imaging experts are supported by the use of standardized image analysis tools and statistical methods. Specifically, the study utilized established software such as Fiji (ImageJ) and MATLAB-R2010a for quantitative image analysis, which are widely accepted in radiological research for their reliability and accuracy.^76,92^

The present work emphasizes that the histograms were generated after selecting ROIs, and the descriptive properties were calculated simultaneously, following standard procedures in image analysis. The statistical measures applied, including sensitivity, specificity, and coefficient of variation, alongside significance testing (ANOVA, Tukey's test), further support the robustness of the results.^93,94^

While the paper states that the data analysis was conducted using these validated software packages and statistical techniques, it does not explicitly mention that independent imaging experts reviewed or validated the results externally. However, the use of recognized analytical tools and rigorous statistical validation lends confidence that the results are reproducible and can be verified by other experts in the field.

According to earlier research, non-receptor-specific clathrin-mediated endocytosis may be used to internalize the Co_3_O_4_ NPs.^95,96^ Similar to this, we discovered in our work that the Co_3_O_4_ NPs have a negative charge on their surface because proteins adsorb after they are resuspended in culture medium, which may help with endocytosis. The primary mechanism of NPs-induced toxicity, once internalized, is the production of ROS.^96,97^ Although ROS are mostly produced in mitochondria in regulated amounts, an excess of them may cause apoptosis and mitochondrial malfunction.^96,98^ The human breast cancer cell line (MCF-7) treated with cobalt nanoparticles has been shown to exhibit defects in several mitochondrial processes, resulting in a weakened mitochondrial membrane potential.^99^ Furthermore, an overall hyperpolarization has been seen as a result of mitochondrial enlargement in eggplant (*Solanum melongena*) induced phytotoxicity by Co_3_O_4_ NPs.^100^ This implies that membrane potential loss was only seen in specific cell types when exposed to Co_3_O_4_ NPs. However, the current literature is limited, and more research is needed before any conclusions can be drawn about Co_3_O_4_ NPs' mode of action.

The different material used in CT guided tools and procedures is Co_3_O_4_ NPs. The use of metallic nanoparticles and their conjugates for CT imaging of tumours is discussed in this work. Co_3_O_4_ NPs have received particular interest. Because CT employs ionising radiation, radiologists strive to keep the dose as low as possible to avoid side effects. The scans are quick in this case, and the radiologist tries to get a clear picture. Both soft and hard tissues can undergo CT scanning. Soft tissue CT imaging requires contrast materials, sometimes referred to as dyes.^101–103^ This advantage is due to the fact that localised therapies guarantee that the tumour cells will be killed specifically while causing little to no harm to healthy tissues. We can use CT imaging to confirm that the tumour has absorbed these treatment drugs.^104–107^

Based on the present work provided, the correlation between histogram shifts in CT images and specific cellular or subcellular changes is supported by existing literature. The observed shifts in gray-level histograms—such as skewness and shifts in peak positions—reflect underlying alterations in tissue density, cellular morphology, and structural integrity caused by Co_3_O_4_ NPs.

Previous studies have demonstrated that cellular exposure to nanoparticles induces morphological and structural changes detectable through imaging techniques. For instance, histopathological and electron microscopy analyses have shown that nanoparticles can cause cell membrane damage, chromatin condensation, apoptosis, and other cellular alterations.^67,96^ These structural changes influence tissue density and composition, which are captured as variations in gray-level distributions in CT images.

Furthermore, histogram analysis has been extensively used to quantify tissue heterogeneity and cellular changes. Xiang *et al.* (2021),^108^ and Wang *et al.* (2022),^91^ highlighted that histogram parameters such as skewness and peak shifts can reliably reflect pathological alterations, including apoptosis and necrosis, at the cellular level. In the context of nanoparticle-induced effects, shifts in histogram peaks are indicative of changes in tissue density corresponding to cell death mechanisms like apoptosis or necrosis, which alter tissue composition and thus CT attenuation values.

Regarding reproducibility and validation, the use of standardized image analysis tools like MATLAB-R2010a and Fiji (ImageJ), along with established statistical measures, enhances the reliability of the findings. These methods are widely accepted in radiological research for quantitative image analysis.^76,92^ Validation by independent imaging experts is crucial, and such approaches are standard practice to ensure the robustness of histograms as biomarkers of cellular changes.

The cytopathic effects of cobalt oxide nanoparticles (Co_3_O_4_ NPs) on Hep-G2 liver cancer cells by quantitative computed tomography (CT) image analysis are investigated. The histogram-based analysis of CT scans provided a non-invasive and reliable method for assessing intracellular changes over time following nanoparticle treatment.

A low mean pixel intensity and mode values, are shown in the control group indicating minimal intracellular density and an absence of nanoparticle production. The results correspond with the expected morphology of untreated Hep-G2 cells, which maintain structural integrity and exhibit no signs of cytotoxic stress. Alarifi *et al.* (2013),^109^ similarly shown that untreated Hep-G2 cells maintain a steady oxidative balance and DNA integrity, serving as a reliable baseline for cytotoxicity assessments.

At 24 hours post-exposure, a notable increase in both mean intensity and mode was detected, signifying the initial uptake of Co_3_O_4_ NPs by the cells. The initial phase of exposure likely generates minor oxidative stress and elicits cytopathic alterations. Mohamed *et al.* (2025),^110^ discovered that Co_3_O_4_ nanoparticles can provoke mitochondrial malfunction and the generation of reactive oxygen species (ROS) within 24 hours, leading to early apoptotic signaling in cancer cells.

Following a 48-hour period, the histogram analysis revealed an increase in mean intensity and mode, accompanied by a significant rise in standard deviation. These alterations signify enhanced nanoparticle accumulation and increased diversity in cellular responses. The findings agreed with previous studies indicating that prolonged exposure to Co_3_O_4_ nanoparticles exacerbates oxidative stress, lipid peroxidation, and DNA damage.^109^

At 72 hours, the peak mean intensity was observed; however, the modal value decreased, and the standard deviation attained its maximum. The reduction in mode, despite heightened mean intensity, may signify the collapse of cellular structures and a loss of uniformity in pixel intensity distribution. These findings are supported by Mohamed *et al.*,^110^ who demonstrated that extended exposure to Co_3_O_4_ nanoparticles triggers p53-independent apoptosis and mitochondrial dysfunction.

The application of red light (655 nm, 50 mW cm^−2^) under photodynamic conditions appears to enhance the cytotoxic effects of Co_3_O_4_ nanoparticles. This interaction resulted from heightened ROS formation upon light activation, as demonstrated by Choi *et al.*,^111^ who found that cobalt-based nanoparticles enhances the effectiveness of photodynamic therapy (PDT) through increased oxidative damage.

The CT-based histogram analysis provided a substantial quantitative approach for evaluating the temporal cytopathic effects of Co_3_O_4_ nanoparticles. The findings highlight the potential of combining nanotechnology with photodynamic methods for targeted cancer therapy, while also underscoring the need for comprehensive evaluation of nanoparticle-induced toxicity.

## 5. Conclusions limitations, and future prespectives

This work offers compelling evidence that Co_3_O_4_ NPs can inhibit the growth of Hep-G2 cell lines. We were successful in creating stable Co_3_O_4_ NPs, with a mean diameter of 27.35 ± 2.0 nm and a diameter range of 21.2 nm to 35.8 nm. According to the SEM imaging study, the synthesized Co_3_O_4_ NPs also looked like bright particles spread out in the nano-range. We got the histograms of each area of interest from the CT images of Co_3_O_4_ NPs for both the control and Hep-G2 groups with Fiji (ImageJ). These show how important our data is. More areas of interest were made with Co_3_O_4_ NPs; different mitochondrial problems were fixed; the potential of the cell membrane went up; and the activity of Hep-G2 cell lines went up. Examinations with an electronic microscope show the changes in cell structure and function that happen whether Co_3_O_4_ NPs are treated or not. Hep-G2 cells die *via* a distinct mechanism involving granule apoptosis. After showing that Co_3_O_4_ NPs have chemical activity, they could also be used in the future as nanomedicine tools. Not only that, but it was discovered that liver cancer cells lost more of their ability to live after they were exposed to a 30 µg mL^−1^ dispersion solution of Co_3_O_4_ NPs that had been heated to 800 °C. We need to deepen their understanding of how cells absorb nanoparticles and the extent to which they absorb Co_3_O_4_ NPs. They also need to do a kinetic analysis or uptake efficiency test on the nanoparticles. We must also examine the cytotoxicity and antiproliferation efficacy in tumor cell lines other than HepG2. Some validation analysis must be performed such as XPS and ICP to confirm the structure of the synthesized Co_3_O_4_ NPs.

## Consent for publication

All authors read and approved the final manuscript.

## Author contributions

MSNE, GSE, and HMA conducted the experiment. MSNE, WMA, GSE, AAE, and MAS analyzed and interpreted the data. All authors contributed in writing and drafting the manuscript. All authors read and approved the final manuscript.

## Conflicts of interest

The authors declare that they have no competing interests.

## Data Availability

The datasets used and analyzed during the current study are available from the corresponding author upon reasonable request.
